# Reductions in smoking progression from 10th to 12th grade across 5 cohorts in Southern California 1995–2014

**DOI:** 10.18332/tid/217624

**Published:** 2026-08-08

**Authors:** Jessica L. Barrington-Trimis, Afton Kechter, Jessica B. Knapp, Feifei Liu, Kiros Berhane, Sandrah Eckel, James Sargent, Rob McConnell

**Affiliations:** 1 Department of Population and Public Health Sciences, University of Southern California, Los Angeles, United States; 2 Department of Biostatistics, Columbia University Mailman School of Public Health, New York, United States; 3 C. Everett Koop Institute, Dartmouth Geisel School of Medicine, Lebanon, United States

**Keywords:** smoking, youth, trends, cigarettes

## Abstract

**INTRODUCTION:**

While initiation of adolescent cigarette smoking has declined in the last two decades, changes to smoking progression have not been examined.

**METHODS:**

Survey data were from five cohorts of youth in Southern California (grade 12 in 1995, 1998, 2001, 2004, or 2014; n=2346). Among those who had smoked a cigarette by grade 10 (n=418), we modeled self-reported past-month, past-year, and lifetime number of cigarettes smoked in grade 12 as a function of grade 10 number of cigarettes and cohort (1995, 1998, 2001, 2004 vs 2014).

**RESULTS:**

Smoking progression declined markedly over time. Among youth who had smoked by grade 10, the 1995 cohort smoked on average 23 times as many lifetime cigarettes by grade 12 as the 2014 cohort (adjusted rate ratio, ARR=22.9; 95% CI: 9.40–55.6). Similar findings were observed for the past year (ARR=30.1; 95% CI: 6.75–134) and the past month (ARR=12.1; 95% CI: 2.19–67.0) number of cigarettes smoked. Over time, females smoked less, but the pattern of decreasing progression in lifetime cigarettes smoked was more pronounced for females than males (lifetime, p for interaction <0.0001).

**CONCLUSIONS:**

The rate of progression among those initiating cigarette smoking in early high school decreased markedly over the two-decade period starting in 1995, suggesting that social or environmental factors may play a substantial role in the escalation of smoking through adolescence.

## INTRODUCTION

Among youth smokers, initial cigarette experimentation has, in the past, rapidly progressed to established regular smoking and the onset of nicotine dependence, especially when smoking is initiated during adolescence and early adulthood^1-3^. Cigarette smoking is associated with substantial adverse health effects and is the leading cause of preventable death in the US^4^. The prevalence of lifetime cigarette smoking among 12th-grade students declined from 64.2% in 1995 to 15.3% in 2025^5^, and, with it, the prevalence of regular cigarette smoking^6^. Much of the decline in regular cigarette smoking – and in dependent nicotine use – (particularly in young adulthood) has been assumed to be driven primarily by decreases in the prevalence of initiation of cigarette smoking over the last several decades^7^. As such, there has been little study of whether the progression from initiation to heavier smoking (independent of the prevalence of initiation and regular cigarette smoking) has changed over this period. Given that there has been no change to the cigarette itself during this time^8^, any changes to progression in the number of cigarettes smoked over time among youth of the same age would necessarily be due to influences outside of the product itself (e.g. social or environmental factors)^4,9^.

In the current study, we sought to determine whether the progression of cigarette smoking during adolescence – following initiation – has changed over the last two decades. We used data from participants of five school-based cohorts who completed secondary school (grade 12) between 1995 and 2014 to assess how patterns of smoking progression evolved over this 20-year time span.

## METHODS

### Study sample

Data are from a large prospective cohort study comprising five successive cohorts of adolescents from communities in Southern California (initially selected to represent diversity in air quality and type of air pollutants) who were recruited in primary school and followed through 12th grade in 1995, 1998, 2001, 2004, or 2014 (hereafter referred to as e.g. ‘1995 cohort’, as appropriate). Recruitment methods and data collection procedures have been described previously^10-13^. The 1995–2004 cohort participants were all recruited from primary schools in the same 12 communities, and were followed yearly through 12th grade; self-reported data on cigarette smoking were collected by questionnaire administered in-person each year, beginning in 4th grade. The 2014 cohort was recruited from 13 communities, of which 12 participated in the 2014 data collection; eight were the same as in the earlier cohorts, and data on cigarette smoking were available in 4 communities. The 2014 cohort was a split cohort (comprising students across two grade levels), which was surveyed every other year (e.g. in 2014, data were collected from both 11th-grade students and 12th-grade students; in 2012, data were collected from 9th and 10th-grade students). In primary analyses, we include data from both splits of the cohort (i.e. those in both 11th and 12th grade in 2014). Sensitivity analyses restricted the sample to only 10th and 12th grade students (removing those who were in 9th/11th grade in the 2014 cohort).

The total analytic sample included 2346 participants who provided data on cigarette smoking in grade 10 of each cohort in the 4 communities common to all cohorts. The analytic sample of individuals among whom progression in the number of cigarettes smoked between 10th and 12th grade was assessed included 418 youth (17.8%) who reported having ever smoked a cigarette by grade 10.

### Ethics statement

This study was approved by the University of Southern California Institutional Review Board, under protocols HS-13-00708 (first approved 26 November 2013), HS-18-00814 (first approved 21 November 2018), HS-18-00706 (first approved 8 November 2018), and HS-12-00180 (first approved 10 May 2012). Student assent and written or verbal parental consent were obtained prior to data collection.

### Cigarette use

Participants were asked to report the number of cigarettes or number of packs of cigarettes that they had smoked in the past month, past year, and in their lifetime. If packs of cigarettes were reported, we multiplied the number of packs by 20 cigarettes (number of cigarettes in a pack) to obtain the total number of cigarettes smoked in a given timeframe (i.e. past month, year, or lifetime).

### Sociodemographic covariates

Participants reported gender (male, female), race/ ethnicity (categorized as non-Hispanic White, Hispanic White, Other), and parental education level (categorized as having a high school degree or lower, some college, a college degree or higher, or missing).

### Statistical analysis

First, we described the analytic sample by cohort, reporting sociodemographic characteristics and the proportion who had ever smoked at least 1 cigarette by grade 10. Next, among those who had ever smoked a cigarette by grade 10, we examined the distribution of the number of cigarettes smoked in the past month, year, and lifetime in grade 10 and grade 12 in each cohort. Because the distributions were highly skewed, we calculated the geometric mean (i.e. multiplicative average, which is less sensitive to large values and data skewed to the right) and standard deviation for each, along with the median and interquartile range. Then, we examined the distribution of the mean change in each participant’s number of cigarettes smoked between grades 10 and 12 in each cohort; similar metrics were used to describe these data.

We then evaluated whether the number of cigarettes smoked in grade 12 differed for older cohorts (compared with the most recent cohort [2014] as the reference group), accounting for the number of cigarettes smoked in grade 10. To statistically model this association, we used negative binomial regression or zero-inflated negative binomial regression (depending on model fit) to model grade 12 cigarette smoking in the past month, year, and lifetime as a function of grade 10 cigarette smoking and cohort, adjusting for gender, race/ethnicity, and parental education level. Final models were selected based on sample size adjusted Bayesian information criterion (BIC) and stability of models. To estimate whether effects differed by gender, we included a product interaction term in each model; if the global interaction test was significant, stratified estimates were reported.

Adjusted rate ratios (ARR, fold-change in grade 12 smoking accounting for grade 10 smoking) for each cohort compared to the 2014 cohort are reported, with 95% confidence intervals (CIs). Analyses were restricted to participants with non-missing data for outcomes in 10th and 12th grade; missing covariate data were handled using a missing indicator approach. *Post hoc* tests were used to evaluate whether effect estimates differed from one another. In sensitivity analyses, we restricted the analytic sample for the 2014 cohort to those who were in 10th and 12th grade for data collection, to ensure robustness of the findings and reduce the likelihood of attenuation of estimates due to inclusion of younger students in the 2014 cohort.

The Statistical Analysis System (SAS, version 9.4) was used for analyses. All hypothesis testing was conducted assuming a two-tailed 0.05 significance level.

## RESULTS

### Sociodemographic characteristics of the sample

Table 1 describes sociodemographic characteristics of the sample. Slightly more participants were female (53.0%), and the sample was largely non-Hispanic White (44.2%) or Hispanic White (40.0%). Parental education level was diverse. There was some variation in the prevalence of these covariates across cohorts. The prevalence of any cigarette smoking by grade 10 decreased substantially over time; in the 1995 cohort, more than 40% of the sample had smoked at least one cigarette by 10th grade (40.8%); this proportion decreased to 33.5% for the 1998 cohort, 27.1% for the 2001 cohort, 12.5% for the 2004 cohort, and 4.1% for the 2014 cohort.

**Table 1:** Demographic characteristics of participants in the analytic sample (by cohort), which includes participants in one of five successive cohorts of adolescents in Southern California who reported any lifetime cigarette smoking by 10th grade (N=2346)

*Characteristics*	*Overall*	*Cohort*				
		*1995*	*1998*	*2001*	*2004*	*2014*
	*n (%)*	*n (%)*	*n (%)*	*n (%)*	*n (%)*	*n (%)*
**Total**, n	2346	294	263	451	401	937
**Gender**						
Male	1102 (47.0)	136 (46.3)	120 (45.6)	212 (47.0)	189 (47.1)	445 (47.5)
Female	1244 (53.0)	158 (53.7)	143 (54.4)	239 (53.0)	212 (52.9)	492 (52.5)
**Race/ethnicity**						
Non-Hispanic White	1036 (44.2)	155 (52.7)	139 (52.9)	238 (52.8)	196 (48.9)	308 (32.9)
Hispanic White	939 (40.0)	83 (28.2)	74 (28.1)	137 (30.4)	143 (35.7)	502 (53.6)
Other	371 (15.8)	56 (19.1)	50 (19.0)	76 (16.8)	62 (15.5)	127 (13.6)
**Parental education level**						
High school or lower	759 (32.4)	101 (34.3)	86 (32.7)	159 (35.3)	106 (26.4)	307 (32.8)
Some college	891 (38.0)	113 (38.4)	99 (37.6)	173 (38.4)	178 (44.4)	328 (35.0)
College degree or higher	581 (24.8)	62 (21.1)	66 (25.1)	104 (23.1)	108 (26.9)	241 (25.7)
Missing	115 (4.9)	18 (6.1)	12 (4.6)	15 (3.3)	9 (2.2)	61 (6.5)
**Smoked cigarette by grade 10**						
No	1928 (82.2)	174 (59.2)	175 (66.5)	329 (72.9)	351 (87.5)	899 (95.9)
Yes	418 (17.8)	120 (40.8)	88 (33.5)	122 (27.1)	50 (12.5)	38 (4.1)

*Frequency of cigarette smoking in grade 10 and grade 12, and change in progression of cigarette smoking from 10th to 12th grade across cohorts*

**Figure 1:**
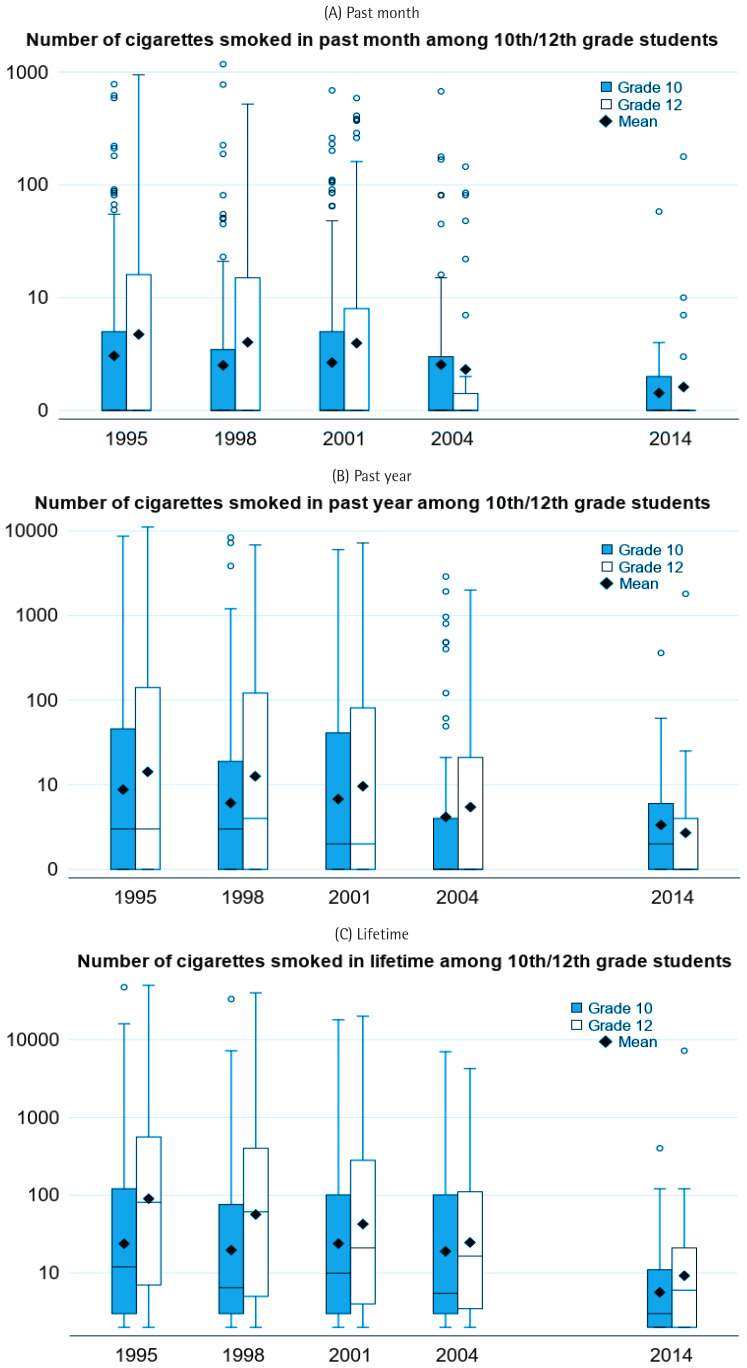
Number of cigarettes smoked in (A) the past month, (B) the past year, and (C) lifetime, in 10th grade and 12th grade, separately by cohort (1993–2014), among participants in one of five successive cohorts of adolescents in Southern California who reported any lifetime cigarette smoking by 10th grade (N=418), using boxplots displaying the median, geometric mean, and interquartile range

**Figure 2:**
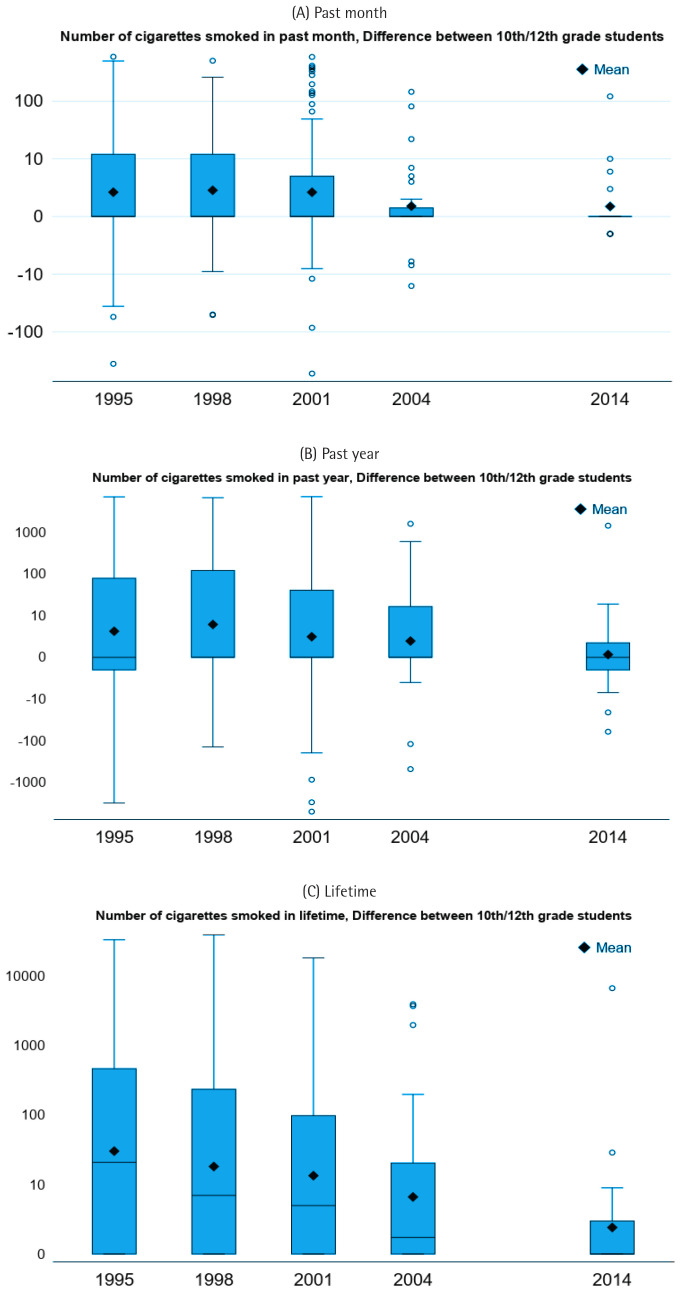
Difference in the number of cigarettes smoked in (A) the past month, (B) the past year, and (C) lifetime, from 10th to 12th grade by cohort (1993–2014), among participants in one of five successive cohorts of adolescents in Southern California who reported any lifetime cigarette smoking by 10th grade (N=418), using boxplots displaying the median, geometric mean, and interquartile range

The mean number of cigarettes smoked in the past month, past year, and lifetime by cohort in 10th grade and 12th grade among those who had ever smoked a cigarette by grade 10 is shown in Figure 1A-1C. Overall, the geometric mean number of cigarettes smoked at each time point decreased substantially over time by cohort. In each cohort, the mean difference (grade 12 - grade 10 for each subject) in the number of cigarettes smoked is displayed in Figure 2A-2C. The increase from 10th to 12th grade declined steadily over time, with smaller increases in progression observed in later cohorts.

*The association of cohort with the change in the number of cigarettes smoked from grade 10 to grade 12*

The number of cigarettes smoked in grade 12 (adjusted for baseline cigarette smoking frequency) declined over time, in models additionally adjusted for gender, race/ethnicity, and parental education level (Table 2). For example, compared to the 2014 cohort, participants in 1995 smoked 12 times as many cigarettes in the past month in grade 12 (ARR=12.1; 95% CI: 2.19–67.0; p=0.004), 30 times as many cigarettes in the past year in grade 12 (ARR=30.1; 95% CI: 6.75–134; p<0.0001), and 23 times as many lifetime cigarettes (ARR=22.9; 95% CI: 9.40–55.6; p<0.0001), though wide confidence intervals resulted in imprecision in true effect estimates. There was a steady decline in lifetime cigarettes smoked in the adjusted model from 1995 to 2004 (after accounting for the number of cigarettes smoked in grade 10). The pattern of change in smoking over time was less clear for past-year and past-month use. It was, however, clear that the more recent cohort had markedly lower levels of progression to more frequent smoking from grade 10 to grade 12 (for lifetime, past year, and past month use) than the earlier cohorts.

**Table 2:** Grade 12 relative increase in cigarettes smoked for each cohort compared with the 2014 cohort in the past month, past year, and lifetime, among participants in one of five successive cohorts of adolescents in Southern California who reported any lifetime cigarette smoking by 10th grade (N=418)

*Item*	*Cohort*	*ARR (95% CI) ^c,d^*	*p*
**Number of cigarettes smoked in the past month^a^**	1995	12.1 (2.19–67.0)^e^	0.004
	1998	7.69 (1.46–40.4)^e^	0.02
	2001	13.8 (2.07–92.6)^e^	0.007
	2004	6.90 (0.84–56.5)^e^	0.07
	2014	Ref	
**Number of cigarettes smoked in the past year^a^**	1995	30.1 (6.75–134)^e^	<0.0001
	1998	29.7 (5.80–152)^e^	<0.0001
	2001	31.1 (5.53–174)^e^	0.0001
	2004	17.2 (2.14–138)^e^	0.008
	2014	Ref	
**Lifetime number of cigarettes smoked^b^**	1995	22.9 (9.40–55.6)^e^	<0.0001
	1998	10.7 (4.33–26.6)^e,f^	<0.0001
	2001	8.92 (3.20–24.8)^e,f^	<0.0001
	2004	6.06 (1.98–18.5)^f^	0.002
	2014	Ref	

Ref: reference group. ARR: adjusted rate ratio.

a Zero inflated negative binomial model.

b Negative binomial model.

c Models are adjusted for gender, race/ethnicity, parental education level, community, and number of cigarettes smoked at baseline in grade 10.

d Estimates sharing the same superscripted letter (e or f), within each timeframe, are not statistically different from one another.

### Gender differences in patterns of cigarette use across cohorts

The interaction of gender by cohort in the adjusted models was statistically significant only for the lifetime number of cigarettes smoked (Table 3). In descriptive analyses, the geometric mean increase in cigarettes smoked from grade 10 to grade 12 was larger in males than in females in all cohorts except the 2001 cohort, albeit with large standard errors. For example, among males, the 1995 cohort had smoked an additional 878 lifetime cigarettes by grade 12, while the 2014 cohort had only smoked an additional 42.3 lifetime cigarettes, reflecting a more than 16-fold difference in the progression from grade 10 to 12 across the cohorts (ARR=16.6; 95% CI: 5.18–52.9). Among females, the 1995 cohort had smoked an additional 232 cigarettes from grade 10 to grade 12, while the 2014 cohort had only smoked an additional 3.3 cigarettes on average, a substantively greater progression for the 1995 (vs 2014) cohort (ARR=59.6; 95% CI: 16.2–219).

**Table 3:** Mean difference in the number of lifetime cigarettes smoked from 10th to 12th grade by cohort and relative increase in cigarettes smoked for each cohort compared with the 2014 cohort, by gender, among participants in one of five successive cohorts of adolescents in Southern California who reported any lifetime cigarette smoking by 10th grade (N=418)

*Cohort*	*Males*		*Females*		*p* *for interaction*
	*MD (SD) ^a^*	*ARR (95% CI) ^b,c^*	*MD (SD) ^a^*	*ARR (95% CI) ^b,c^*	
1995	878 (3827)	16.6 (5.18–52.9)^d^	232 (1006)	59.6 (16.2–219)^d^	<0.0001
1998	761 (4722)	15.8 (4.69–53.2)^d^	245 (1488)	29.1 (8.68–97.6)^d^	
2001	191 (1651)	2.72 (0.72–10.3)^e^	350 (1744)	44.1 (13.2–148)^d^	
2004	175 (838.8)	13.0 (2.83–59.7)^d^	41.8 (473)	3.25 (0.76–13.9)^e^	
2014	42.3 (432.8)	Ref	3.3 (36.0)	Ref	

Ref: reference group. MD: mean difference. SD: standard deviation. ARR: adjusted rate ratio.

a Mean difference in the number of lifetime cigarettes smoked from 10th grade to 12th grade.

b Estimates are from negative binomial models, adjusted for gender, race/ethnicity, parental education level, community, and number of cigarettes smoked at baseline in grade 10.

c Estimates sharing the same superscripted letter (d or e), by gender, are not statistically different from one another.

### Sensitivity analyses

We observed similar patterns of progression from grade 9 to grade 11 and from grade 10 to grade 12 in the split 2014 cohort, with only slightly greater increases from grade 10 to grade 12. Results are unlikely to be substantively altered by the inclusion of the lower split.

## DISCUSSION

The progression to higher levels of cigarette smoking from grade 10 to grade 12 was markedly attenuated from 1995 to 2014 across five prospectively followed adolescent cohorts, with a pattern of decline in progression across two decades most apparent for lifetime cigarette use. In adjusted models, the earliest cohort (1995) smoked 23 times as many lifetime cigarettes as the most recent cohort (2014) in grade 12, adjusting for grade 10. The adjusted pattern of decline over time for past-year and past-month use was less clear (estimates with wide confidence intervals). However, these models demonstrated that the most recent cohort progressed to more frequent smoking at a lower rate than each of the other cohorts.

These results are good news, as they suggest that the well-known decline in prevalence of cigarette use over two decades^4^ was accompanied in these communities by considerably smaller increases in smoking during a key developmental period^9^ and markedly lower levels of use in grade 12. The results may also have implications for the continued relevance of studies showing that more than two-thirds of those who initiated cigarette smoking subsequently became regular smokers by early adulthood, driven by nicotine dependence^1^. Over the last 25 years, very little has changed in the composition of cigarettes, including in the levels of nicotine – the addictive chemical in cigarettes^8^. Historically, it was thought that the risk of progression to more frequent smoking or nicotine dependence was due primarily to the addictive properties of nicotine^8^. Some individuals have reported that they felt ‘addicted’ to nicotine (or screened positive for nicotine dependence on quantitative surveys) after only one or two cigarettes^14-16^; those progressing early are likely doing so at least in part due to nicotine dependence and the physiological need to satiate nicotine cravings. However, since the level of nicotine in cigarettes has remained stable over the last 20 years^8^, changes in the rate of progression from grade 10 to 12 among those who have initiated by grade 10 is unlikely to be driven by changes in the level of nicotine (i.e. this lower rate of progression observed in the 2014 cohort does not correspond to a reduction in nicotine level in cigarettes).

The lower rate of progression from grade 10 to 12 and the level of smoking by grade 12 observed in the more recent cohorts is likely due to social or environmental changes in the way that cigarettes are viewed and accepted within the adolescent population^4,9^. The appeal of the cigarette to youth who have already initiated smoking (even if only one cigarette) is likely a combination of the product itself [including the taste, flavor, and physiological effects of nicotine (e.g. a nicotine ‘buzz’, satisfaction of cravings, etc.)] and how smoking cigarettes is perceived^9^. If the product itself is appealing, or if the act of smoking cigarettes (i.e. being seen as a ‘smoker’) is appealing, adolescents are more likely to continue smoking and increase their use over time. The decrease in progression in the number of cigarettes smoked in more recent years is unlikely to have resulted from changes in the appeal of the product itself (as the product has not changed over this time). We may suggest that the appeal of smoking has more to do with the acceptance of smoking within this population, which has decreased over time. In California, in particular, cigarette smoking has become widely stigmatized, and the stigma associated with smoking has increased over time^17-19^. Cigarettes are simply less appealing to youth. The reduced appeal of cigarettes (and increased stigmatization) may explain why adolescents experimenting with cigarette smoking in grade 10 in the most recent cohort progressed only modestly over the next two years of high school. This may also be relevant to the low rates of progression in females. Females may be more susceptible to the stigma associated with cigarette smoking and may be more likely to discontinue use or smoke less frequently to avoid such stigma^20^. These results are promising for continued efforts to reduce smoking among young people. Even if youth begin to experiment with cigarettes early, efforts to reduce the social, environmental, or psychological appeal of smoking (much of which is already happening) can have a greater impact on reductions in progression to regular smoking and eventual development of dependence than immediate efforts to address early dependence (which may be less of a driving factor, and which is generally harder to impact).

The evolving tobacco landscape has created rapidly changing patterns and trends of use, which may have an indirect impact on the findings observed herein. Specifically, with more options for nicotine use, youth may have different product-type initiation patterns (e.g. groups of youth who tried cigarettes first versus e-cigarettes first, or use only e-cigarettes versus cigarettes) that may affect progression^7^. The markedly lower levels of smoking progression in 2014 could possibly be affected by these changes. However, this most recent cohort was followed from 10th grade in 2012 (to 12th grade in 2014), largely preceding the recent epidemic of e-cigarette use^21^. Future research is needed to examine how the changing tobacco product landscape may impact the risk of progression following experimentation. Given the differences between cigarettes and e-cigarettes^9,21^, additional research is needed to understand any divergence in progression risk, which will be important to ongoing efforts to reduce nicotine use in young people.

### Limitations

This study is subject to some limitations. The five cohorts were not evenly spaced over the 20 years of interest, with a 10-year gap between the most recent two cohorts (2004–2014). Southern California is a geographical region with low rates of cigarette smoking in adolescents and young adults; as such, results may not generalize to other geographical regions. Data on the number of cigarettes smoked at each time point were self-reported and could introduce information bias, which may attenuate findings (e.g. if youth under-reported the number of cigarettes smoked due to perceived social norms, particularly in more recent years when smoking had become less socially acceptable). Given the observational nature of the data collected, causality cannot be determined. Because we were able to examine the number of cigarettes smoked prospectively between grade 10 and grade 12 in only 4 communities common to all 5 cohorts of the CHS, the current study had a modest sample size and estimates with wide confidence intervals. Nevertheless, there were some clear patterns of decreasing smoking progression over the years of this study. We were unable to examine the use of other nicotine products (e.g. little cigars, hookah) as these data were not available in the older cohorts or in the 2014 cohort in grade 10. E-cigarette use also was not available in grade 10 in the 2014 cohort; thus, we could not investigate the impact of e-cigarette use on cigarette progression. No data were available on other potential confounders (e.g. mental health), which could lead to residual confounding. This sample was restricted to high school students; data on progression in smoking frequency beyond high school could not be evaluated. Given recent evidence of a later age of transition to regular smoking in more recent years, additional studies examining whether these patterns extend into early adulthood are warranted.

## CONCLUSIONS

The progression and rapid escalation in cigarette smoking during a key developmental period from grade 10 to 12 was markedly reduced during two decades ending in 2014, with particularly large reductions in the most recent cohort. Further research to assess the generalizability and to understand the reasons for this promising trend is needed to continue to develop prevention policies for youth.

## Data Availability

The data supporting this research are available from the authors on reasonable request.
